# 

**Published:** 1860-01

**Authors:** 


### RECEIPTS FOR THE JOURNAL UP TO JANUARY 1st, 1860.

G. V. Ewing, Ill., Vol. 3–1860, .......... $2

A. A. & H. C. Rawson, Ill., Vol. 3–1860, .......... 2

Dr. Hiram Duncan, Ind., Vol. 2–1859, .......... 2

J. F. Hoge, Ill., Vol. 3–1860, .......... 2

T. Newkirk, Ind., Vol. 2–1859, .......... 2

Wm. S. Robertson, Iowa, Vol. 3–1860, .......... 2

Henry Warmouth, Ill., Vol. 3–1860, .......... $2

T. B. Dever, Ill., Vol. 3–1860, .......... 2

J. B. Cloud, Ill., Vol. 3–1860, .......... 2

Geo. W. Slack, Ind., Vol. 2–1859, .......... 2

Hiram Hall, Ill., Vol. 3–1860, .......... 2

Coe & Constance, Ind., Vol. 2–1859, .......... 2

